# Predictive value of glycoprotein DKK3 for early neurological deterioration after ischemic stroke

**DOI:** 10.1016/j.clinsp.2024.100360

**Published:** 2024-04-27

**Authors:** DongLiang Zhou, HongWei Qin, Lei Miao, Ying Xu, Lan Yu, JianMin Wang

**Affiliations:** Department of Neurology, Renhe Hospital of Baoshan District, Shanghai City, China

**Keywords:** Acute ischemic stroke, Early neurological deterioration, Dickkopf-3, Predictive value, Risk of death

## Abstract

**Objective:**

To explore the value of serum Dickkopf-3 (sDKK3) in predicting Early Neurological Deterioration (END) and in-hospital adverse outcomes in acute ischemic stroke (AIS) patients.

**Methods:**

AIS patients (*n* = 200) were included and assessed by the National Institutes of Health Stroke Rating Scale. Serum Dkk3 levels were assessed by ELISA. END was defined as an increase of ≥ 4 points in NIHSS score within 72h. The biological threshold of sDKK3 level and END occurrence were predicted based on X-tile software. Primary outcomes were END and all-cause death, and the secondary outcome was ICU admission during hospitalization. The logistic regression model and Cox risk regression model were applied to evaluate the relationship between DKK3 level and END incidence, all-cause in-hospital mortality, and in-hospital adverse outcomes (ICU admission).

**Results:**

During hospitalization, the incidence of END in patients with AIS was 13.0 %, and the mortality rate within 7 days after END was 11.54 % (3/26). In patients below the serum DKK3 cutoff (93.0 pg/mL), the incidence of END was 43.5 % (20/48). Patients with lower sDKK3 levels were associated with a 1.188-fold increased risk of developing END (OR = 1.188, 95 % CI 1.055‒1.369, *p* < 0.0001). However, there was no significant association with admission to the ICU. sDKK3 below the threshold (93.0 pg/mL) was a risk factor for death.

**Conclusion:**

Predictive threshold levels of serum DKK3 based on X-tile software may be a potential predictive biomarker of in-hospital END in patients with AIS, and low levels of DKK3 are independently associated with increased in-hospital mortality.

## Highlights

• Analysis of clinical characteristics of subjects.
• DKK3 levels are associated with END in patients with AIS.
• DKK3 levels are associated with in-hospital death in patients with AIS.

## Introduction

According to the 2021 epidemiological study, stroke is the second leading cause of disability and mortality worldwide, and Acute Ischemic Stroke (AIS) accounts for about 80 % of all strokes [1]. In China, 2,000,000 new stroke cases are diagnosed each year, and stroke-related deaths account for 20 % of the total mortality [2]. Ischemic stroke caused by vascular occlusion is often serious and has a poor prognosis [3]. Hemodynamic changes after stroke, bleeding abnormalities, inadequate collateral circulation, or pre-existing comorbidities are all potential causes associated with Early Neurological Deterioration (END) [4]. In a meta-analysis, the incidence of post-stroke END was 13.8 % [5]. In the study of Lee SeungJae et al., the post-stroke END rate was 19.0 % [6]. Most studies to date have used the National Institutes of Health Stroke Score (NIHSS) 7, 8, 9, 10. Stevenson Claire D et al. showed that the incidence of END after AIS was 8.0 %, and the occurrence of END was associated with higher mortality within 7 days of the patient [10]. The study of Fan JuSing et al. also showed that END was associated with 1-week and 30-day mortality and poor neurological prognosis upon discharge [11]. Therefore, improving the prognosis of patients with progressive stroke is a clinical path to alleviate the burden of stroke disease in China. Therefore, by identifying the risk of END and based on the principles of early diagnosis and treatment and individualized treatment, early intervention is of great significance in improving the prognosis of patients. However, the clinical prediction of END of AIS patients is few, and the prediction is mainly based on the imaging findings in the past, which is likely to lead to diagnosis and treatment delay. Therefore, the development of early diagnostic molecular markers of END has clinical significance for improving patient prognosis.

Dickkopf3 (DKK3) is a secreted glycoprotein that is highly expressed in vascular endothelial cells and muscle cells and mainly regulates the Wnt/β-catenin pathway [9,12]. DKK3 was originally considered as a biomarker for tumors [13]. However, recent studies have found that DKK3 can prevent cardiac dysfunction and ventricular remodeling after myocardial infarction [14], and can antagonize the atherosclerosis process by regulating inflammatory response and Wnt/β pathway [15]. In addition, in animal studies, the severity of atherosclerosis in large vessels was negatively correlated with DKK3 expression [16]. All the above evidence suggests that DKK3 can predict the pathological course of human atherosclerosis and is closely related to cardiovascular diseases. A recent cohort study showed that DKK3 is widely distributed and highly expressed in neurons in multiple brain regions, especially in the central nervous system, where it is involved in basic neuronal processes [17]. Since the END event after ischemic stroke is closely related to the degree of cerebrovascular atherosclerosis and intervention time window, metabolic abnormalities caused by ischemia lead to increased nerve cell injury and death. Therefore, it is hypothesized that decreased DKK3 levels in patients after ischemic stroke may be a risk factor for poor prognosis, and therefore detection of DKK3 levels at admission can predict the adverse outcomes in patients.

This study was based on X-tile software (28578146) to predict the biological threshold of DKK3 serum concentration level and END and to explore the predictive value of serum DKK3 level for END and in-hospital adverse events.

## Materials and methods

### Study participants

From October 2020 to June 2022, 200 patients admitted to the neurology ward of Renhe Hospital of Baoshan District with a definite diagnosis of AIS were eventually included. Thirty-two healthy control subjects (aged 45‒85 years) were from the health management Center of Renhe Hospital of Baoshan District. This research was approved by the Renhe Hospital of Baoshan District (Approval number: 201912SH24) Institutional Review Committee and Ethics Committee. All participants received and signed informed consent. The study follows the Standards for Reporting of Diagnostic Accuracy (STARD) reporting guideline.

Inclusion criteria:

1) Age ≥18 years old;

2) The time window between symptom recognition and admission does not exceed 24 hours;

3) The diagnosis of AIS meets the World Health Organization criteria [18].

Exclusion criteria:

1) Patients with previous stroke history;

2) Patients with combined with atrial fibrillation;

3) Patients receiving interventional therapy;

4) Patients with intracranial hemorrhagic disease;

5) Patients with severe hepatic and renal insufficiency;

6) Patients with serious stress conditions such as urinary system infection;

7) Patients with vital organ failure, malignant tumors;

8) Patients can not cooperate to complete all checks.

### Blood sample collection

All subjects were fasted overnight to take blood samples, which were processed within 2 hours. To obtain plasma and serum, blood was collected into test tubes containing EDTA, heparin, or without anticoagulants. The samples were centrifuged at 1300g at 4°C for 20 min and stored at -80°C.

### Clinical features and laboratory tests

Clinical features and medical records were reviewed by anthropometry during clinical visits. Clinical information included age, gender, smoking (≥ 5 cigarettes/day for more than 3 years), alcohol consumption (> 50 g/day for more than 3 years), history of hypertension, diabetes, Myocardial Infarction (MI), lesion site of stroke, NIHSS score at admission, brachial artery pressure at admission, Fasting Blood Glucose (FBG), Homocysteine (HCY), serum Total Cholesterol (TC), Triglyceride (TG), High-Density Lipoprotein C (HDL-C), Low-Density Lipoprotein C (LDL-C), and stroke subtype.

Auxiliary examinations included CT, CTA, multimodal MRI, electrocardiogram, cardiac ultrasound, vascular ultrasound, etc. In anterior circulation ischemic stroke, macrovascular disease is defined as either carotid ultrasound showing an occlusion of extracranial internal carotid artery on the same side of the lesion, or magnetic resonance angiography showing a near occlusion of intracranial internal carotid artery or middle cerebral artery M1 segment. Two experienced senior neurologists evaluated the clinical manifestations and brain images and classified cerebral infarction as either anterior or posterior circulatory ischemic stroke.

Samples were tested by the hospital's central laboratory: An automatic analyzer Beckman Coulter AU5800 (Beckman Coulter) measured FBG, HCY, TC, TG, HDL-C, and LDL-C.

Serum DKK3 (Abcam, ab236710) levels expressed in picograms per milliliter (pg/mL) were measured. All samples were repeated in one assay to avoid interassay variation. ELISA measured less than 3 % intra-assay variation with a detection sensitivity of 34.1 pg/mL.

### Outcome events

END was defined as the increase of 4 points in the total NIHSS score within 72 hours after admission [19], and all-cause death was defined as the all-cause death during admission. A secondary outcome event, ICU admission, was defined as a patient's transfer to the ICU during admission due to other sudden illness or illness deterioration.

### sDKK3 biological threshold

DKK3 values as well as the main event were input into X-tile software (https://medicine.yale.edu/lab/rimm/research/software/; Version: V3.6.1). The software automatically determines the optimal biological threshold based on the enumeration method. Since the biological threshold needs to be established as an integer clinically, it is necessary to determine the two integer adjacent values above and below the biological threshold.

### Data analysis

Continuous variable data are expressed as median (25^th^ and 75^th^ percentiles), while categorical variables as frequency ( %). Shapiro-Wilk analysis tested the normality of the data. Variables in normal distribution were compared by independent Student's *t*-test. The two sets of skew distribution variables were compared using the Mann-Whitney *U* test, followed by Bonferroni correction. Count or categorical variables were analyzed by Person Chi-Square or Fisher exact test. Unsupervised Principal Component Analysis (PCA) explored the dimensionality reduction of clinical features to obtain a small number of variables through linear changes from multiple variables. Multicollinearity was determined by collinearity diagnosis in linear regression, with VIF values less than 5 indicating no serious multicollinearity between variables. Stepwise backward multivariate Logistic regression (likelihood ratio) screened factors associated with END and ICU admission. Univariate and multivariate Cox risk regression analyses examined the factors affecting all-cause mortality. Furthermore, the construction of Receptor Operating Characteristic (ROC) curves was employed to assess the predictive ability of serum DKKs in identifying END events in patients with AIS). SPSS software 22.0 was utilized for analysis and GraphPad Prism 9.5.0 for mapping; *p <* 0.05 was considered statistically significant.

## Results

### Characteristics of participants with END or without END

As shown in Table 1, the study populations were grouped according to the definition of END and the baseline characteristics. The incidence of END was 13.0 % (26/200). As can be seen from Table 1, the median age of END patients was 69 years (IQR 63‒72), which was higher than the median age of non-END patients, 62 years (IQR 54‒69, (*p* = 0.0001). Of note, the NIHSS score at admission was higher in END patients than in non-END patients (*p =* 0.0001). In addition, END patients had a relatively high history of diabetes, SBP, FBG, HCY, TC, TG, and LDL-C at admission. No differences were observed between the two groups in gender, smokers, drinkers, history of hypertension, history of myocardial infarction, site of stroke lesion, and stroke subtype (p > 0.05).

**Table 1:** Characteristics of participants with END or without END.

Characteristics	END (*n =* 26)	Non-END (*n =* 174)	*p*-value
Age, years	69 [63‒72]	62 [54‒69]	0.0001
Gender, *n* (%)			
Male	18 (69.2)	104 (59.8)	0.356
Female	8 (30.8)	70 (40.2)	
Smoking, *n* (%)	4 (15.4)	29 (16.7)	1
Drinking, *n* (%)	7 (26.9)	36 (20.7)	0.471
Medical history, *n* (%)			
Hypertension	10 (38.5)	60 (34.5)	0.692
Diabetes	9 (34.6)	30 (17.2)	0.037
MI	1 (3.8)	32 (18.4)	0.086
Lesion location, *n* (%)			0.484
Anterior cerebral circulation	20 (76.9)	119 (67.6)	/
Posterior cerebral circulation	6 (23.1)	51 (29.0)	/
Both	0 (0.0)	6 (3.4)	/
NIHSS score of admission	10 [8‒12.5]	7[5‒9]	0.0001
SBP, mmHg	161 [142‒170]	150 [135‒163]	0.0006
DBP, mmHg	88 [78‒90]	87 [79‒92]	0.4925
FBG, mmoL/L	7.7 [6.5‒9.3]	6.7 [5‒8]	0.0001
HCY, µmoL/L	20.0 [13.2‒25.0]	16 [12.0‒20.1]	0.0028
TC, mmoL/L	5.7 [5.0‒6.1]	5.2 [4.6‒5.8]	0.0172
TG, mmoL/L	1.4 [0.98‒2.2]	1.6 [1.2‒2.3]	0.234
HDL-C, mmoL/L	1.45 [1.36‒1.53]	1.26 [1.15‒1.45]	0.0001
LDL-C, mmoL/L	3.45 [2.84‒4.03]	3.00 [2.50‒3.41]	0.0256
Stroke subtype, *n* (%)			0.979
Large artery atherosclerosis	12 (46.1)	83 (47.2)	/
Small vessel occlusion	4 (15.4)	30 (17.0)	/
Cardioembolism	4 (15.4)	22 (12.5)	/
Undetermined	6 (23.1)	41 (23.3)	/

NHISS, The National Institutes of Health Stroke Scale; SBP, Systolic Blood Pressure; DBP, Diastolic Blood Pressure; CHD, Coronary Heart Disease; MI, Myocardial Infarction; AF, Atrial Fibrillation; FBG, Fasting Blood Glucose; HCY, Homocysteine; TC, Total Cholesterol; LDL-C, Low Density Lipoprotein Cholesterol, TG, Triglycerides; HDL-C, High-Density Lipoprotein Cholesterol; END, Early Neurological Deterioration (NHISS increased by ≥4 points 72 hours after admission). Variables with a skewed distribution were reported as the median and IQR and compared using Mann-Whitney *U* test; The Chi-Square test was used for categorical variables, and categorical variables are presented as percentages and frequencies.

### DKK3 levels are associated with END in patients with AIS

**Fig. 1:**
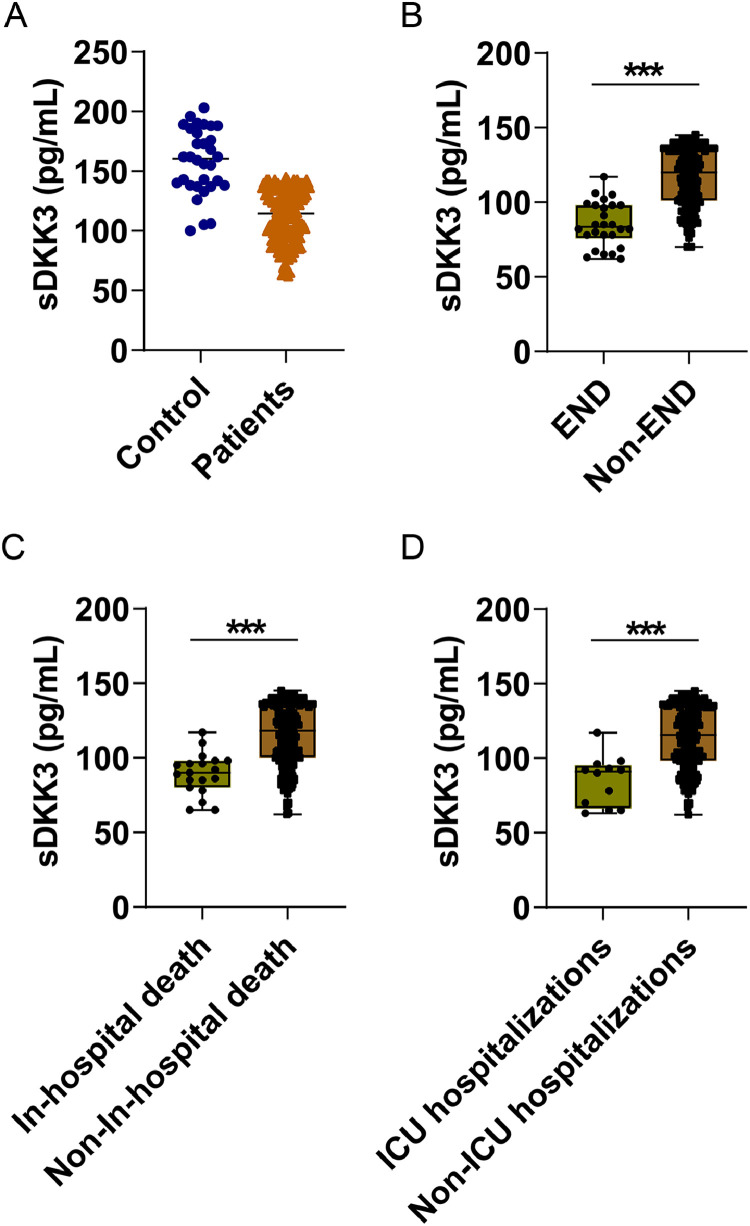
Serum DKK3 levels in (A) healthy people and AIS patients; (B) patients with END and patients without END; (C) Patients who died during their stay (*n =* 19) and those who did not (*n =* 181); (D) patients admitted to the ICU during their stay and those not admitted to the ICU.

**Fig. 2:**
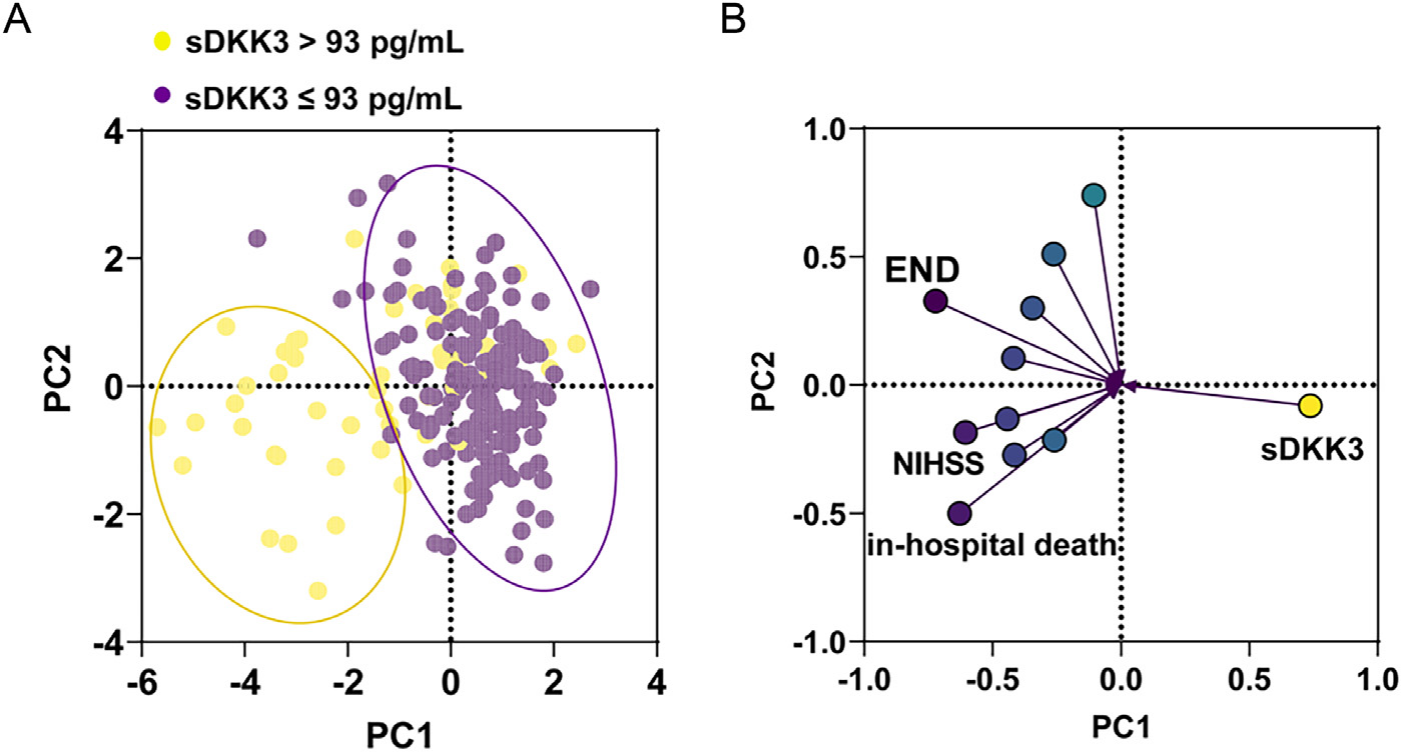
Unsupervised principal component analysis (A) and load analysis (B) of serum DKK3 levels and clinical features.

sDKK3 levels in AIS patients were lower than those in healthy controls (*p <* 0.001, Fig. 1A). In addition, there were lower sDKK3 levels in END compared to Non-END patients (*p <* 0.001) (Fig. 1B). Similarly, sDKK3 levels were also lower in patients who died and those who entered IUC (*p <* 0.001) (Fig. 1B‒C). The authors took END as the predicted event and obtained the biological threshold of sDKK3 as 93.0 pg/mL through X-tile software. The median length of stay was 16 days and the longest was 87 days. All-cause death and ICU admission in AIS patients were followed up, and deaths occurred during hospitalization in 19 cases (9.5 %) and ICU admission in 14 cases (7.0 %). Next, to further observe the relationship between sDKK3 and clinical features, an unsupervised PCA analysis of sDKK3 was performed including variables with statistical differences. Individuals in the same group tended to cluster, and fewer individuals overlapped between the two groups (Fig. 2A). Fig. 2B shows the load coefficients of various factors, among which the variables with a higher contribution rate (absolute load coefficient greater than 0.6) in principal component 1 included NIHSS at admission, END, and death during hospitalization. This further suggests that reduced sDKK3 levels may be associated with an increased risk of END and in-hospital death.

Next, stepwise backward Logistic regression analysis found that the risk of END and ICU admission in patients older than 75 years was 4.688 times that of patients younger than 75 years (OR = 4.688, 95 % CI 1.619‒7.431, *p <* 0.001) and 1.094 times (OR = 1.094, 95 % CI 1.007‒1.188, *p =* 0.034). Patients with a history of hypertension had a 1.283-fold increased risk of entering IUC (OR = 1.283, 95 % CI 1.104‒2.598, *p =* 0.001). NIHSS score at admission was a risk factor for END and IUC (OR=1.249, 95 % CI 1.054‒1.483, *p =* 0.011; OR = 1.429, 95 % CI 1.142‒1.788, *p =* 0.002). Notably, sDKK3 below the threshold (93.0 pg/mL) was associated with an increased risk of END (OR = 1.188, 95 % CI 1.055‒1.369, *p <* 0.0001). In addition, FGB and HCY were risk factors for END in patients (OR = 1.072, 95 % CI 1.059‒1.365, *p <* 0.0001; OR = 1.311, 95 % CI 1.125‒1.772, *p =* 0.012), while HCY was also a risk factor for admission to ICU (OR = 1.170, 95 % CI 1.096‒1.226, *p =* 0.029) (Table 2, Table 3).

**Table 2:** Logistic analysis of factors related to the occurrence of END in patients.

Items	β	S.E	Wald	OR	95 % CI	*p-value*
Age (years)						
< 75	Ref					
≥ 75	0.066	0.33	4.005	4.688	1.619‒7.431	<0.0001
sDKK3 (ng/mL)						
≥ 93	Ref					
< 93	0.205	0.096	14.353	1.188	1.055‒1.369	<0.0001
NIHSS score of admission	0.222	0.088	6.41	1.249	1.054‒1.483	0.011
FBG	1.112	0.285	15.554	1.072	1.059‒1.365	<0.0001
HCY	0.069	0.464	6.341	1.311	1.125‒1.772	0.012

OR, Odds Ratio; CI, Confidence Interval; NIHSS, National Institutes of Health Stroke Scale.

Age, gender, smoking, alcohol consumption, history of hypertension, history of diabetes, sDKK3 baseline, NIHSS score, Systolic Blood Pressure (SBP), Diastolic Blood Pressure (DBP), Fasting Blood Glucose (FBG), Homocysteine (HCY), Serum Total Cholesterol (TC), Serum Triglyceride (TG), High Density Lipoprotein-C (HDL-C), and Low Density Lipoprotein-C (LDL-C) were included.

**Table 3:** Logistic stepwise backward analysis of the relevant analysis factors of patients entering ICU.

Items	β	S.E	Wald	OR	95 % CI	*p-value*
Age (years)						
< 75	Ref					
≥ 75	0.09	0.042	4.472	1.094	1.007‒1.188	0.034
Hypertension						
No	Ref					
Yes	0.249	0.077	10.557	1.283	1.104‒2.598	0.001
NIHSS score of admission	0.357	0.114	9.754	1.429	1.142‒1.788	0.002
HCY	0.092	0.057	2.985	1.17	1.096‒1.226	0.029

OR, Odds Ratio; CI, Confidence Interval; NIHSS, National Institutes of Health Stroke Scale; END, Early Neurological Deterioration.

Age, gender, smoking, alcohol consumption, history of hypertension, history of diabetes, sDKK3 baseline, NIHSS score, Systolic blood Pressure (SBP), Diastolic Blood Pressure (DBP), Fasting Blood Glucose (FBG), Homocysteine (HCY), Serum Total Cholesterol (TC), Serum Triglyceride (TG), High Density Lipoprotein-C (HDL-C), Low Density Lipoprotein-C (LDL-C), END event occurred within 72h after admission were included.

### DKK3 levels are associated with in-hospital death in patients with AIS

**Fig. 3:**
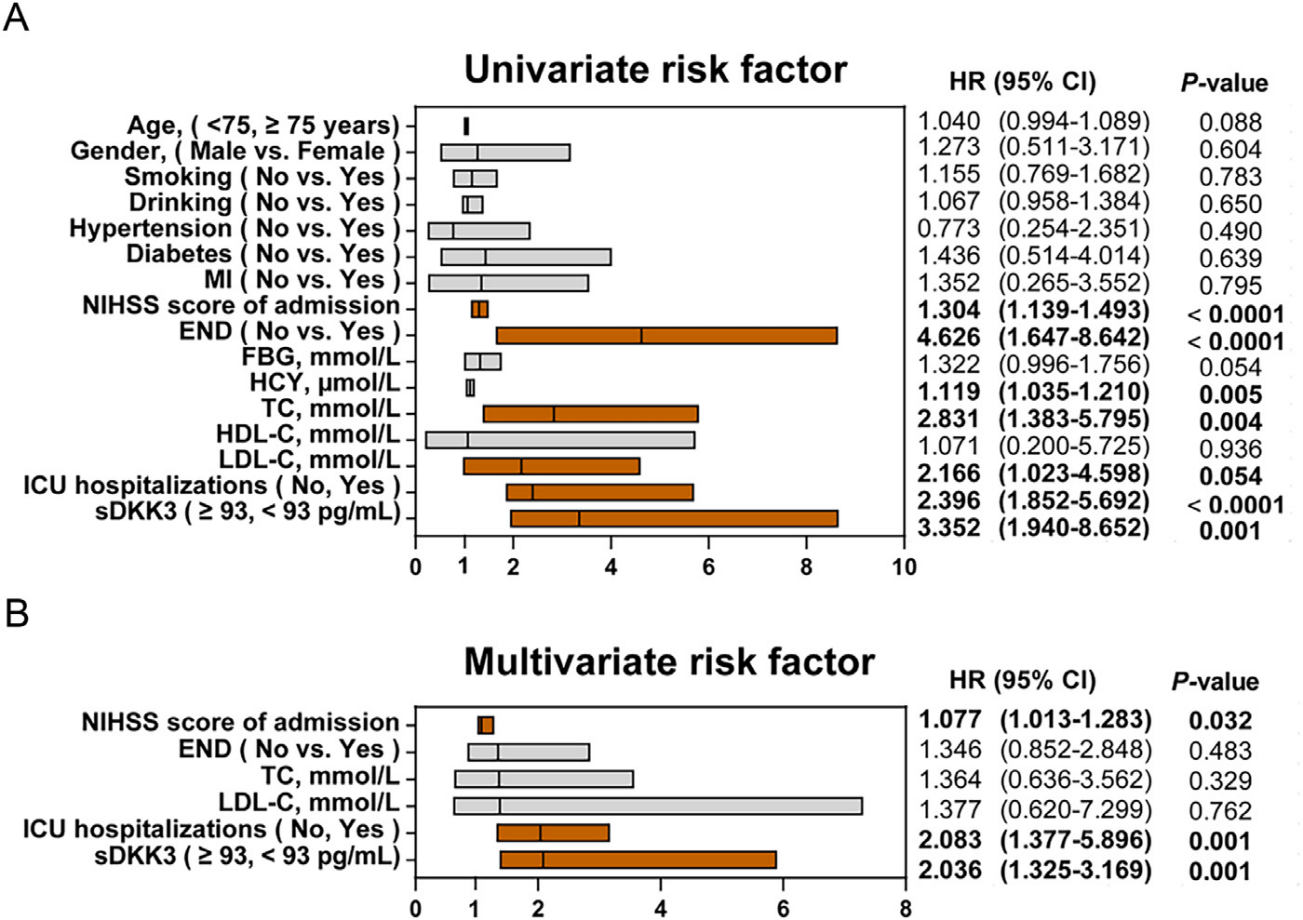
Factors associated with all-cause death during hospitalization in univariate (A) and multivariate (B) Cox risk regression analyses.

**Fig. 4:**
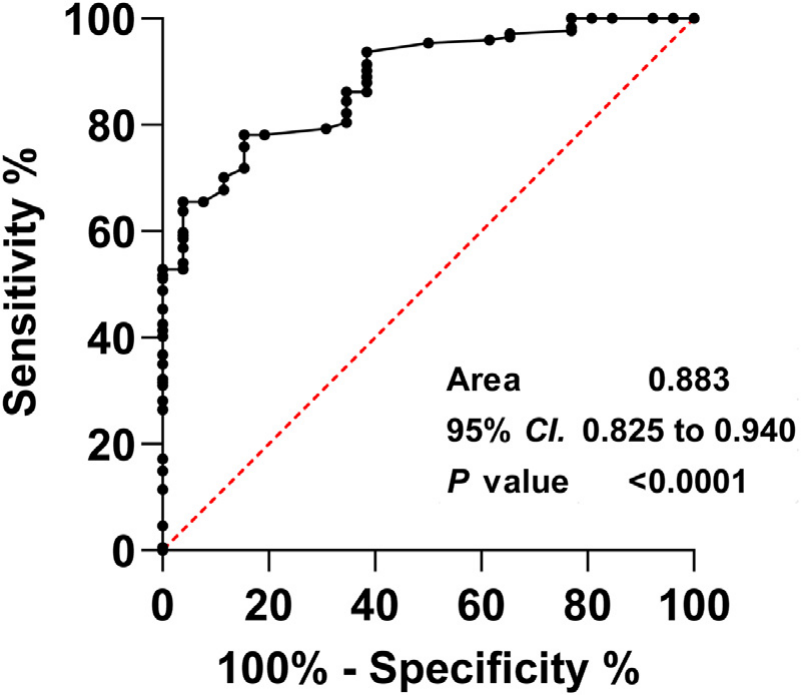
The construction of Receptor Operating Characteristic (ROC) curves was employed to assess the predictive ability of serum DKKs in identifying END events in patients with AIS).

Univariate and multivariate Cox risk regression analysis determined that NIHSS score, END occurrence, TC, LDL-C, ICU admission, and sDKK3 below the threshold (93.0 pg/mL) at admission were risk factors for in-hospital death (Fig. 3A). NIHSS was an independent risk factor for death of patients at admission, and the risk increased by 1.077 times for every 1-point increase (HR = 1.077, 95 % CI 1.013‒1.283, *p =* 0.032). Patients admitted to IUC had a 2.083-fold increased risk of death (HR = 2.083, 95 % CI 1.377‒5.896, *p =* 0.001). sDKK3 below the threshold (93.0 pg/mL) was an independent risk factor for death (HR = 2.036, 95 % CI 1.325‒3.169, *p =* 0.001). In the present study, it was determined that the level of sDKK3 can effectively predict the occurrence of END in AIS, as evidenced by the analysis of the ROC curve. The identified cutoff value for sDKK3 was found to be 97.6 pg/mL, with corresponding specificity and sensitivity values of 78.16 % and 84.64 % respectively (Fig. 4). Notably, this threshold closely aligns with the predictive threshold for in-hospital mortality in patients utilizing X-title software.

## Discussion

Little research has been done to predict END, especially in patients with AIS. This prospective study reported that patients with lower sDKK3 values (< 93 pg/mL) had a significantly higher risk of developing END. The authors can assume that patients whose condition improves or stabilizes may differ from those whose condition worsens in terms of sDKK3 levels. Further analysis showed that sDKK3 levels were lower in AIS patients compared to healthy control groups. Results showed that sDKK3 < 93 pg/mL was significantly associated with END and in-hospital death in AIS patients, although this was not associated with the adverse event of admission to IUC. Previous studies have shown that individuals with reduced DKK3 levels have a higher risk of developing breast cancer [20], ovarian cancer [21], malignant glioma [22], and pancreatic cancer [13]. In the past few years, emerging research has focused on low levels of DKK3 in cardiovascular adverse events, and most studies have suggested that the mechanism involves both classical and non-classical Wnt pathways. In addition, DKK3 regulates the JNK p38 signal by negatively regulating apoptosis signals to prevent myocardial infarct-induced cardiac remodeling [14]. According to the literature, there have been no prospective studies on the relationship between sDKK3 levels and END. The present findings highlight low sDKK3 levels as an independent predictor of early neurological changes, particularly END in AIS.

In patients with AIS, END itself was a risk factor for poor prognosis. It has been reported that END patients have a 30-fold increased risk of future disability compared with non-END patients [23]. END is a complex pathological change affected by multiple mechanisms and environments [24]. Therefore, identifying these patients facilitates the prevention of adverse outcomes.

According to epidemiological studies and related studies, a baseline stroke scale can predict the risk of END in patients with AIS [25]. In this study, variables with statistical differences were included through PCA analysis. NIHSS at admission, END, and death during hospitalization were screened as correlated with sDKK3 levels. Further, this study showed that low levels of sDKK3 were significantly associated with END in AIS patients. In addition, the risk of END and ICU admission was 4.688 times higher in patients older than 75 years. Similar to the findings of Ibanez and Laura et al., a history of hypertension is also a risk factor for patients entering the ICU [26]. In addition, many studies have shown that higher NIHSS at admission is significantly associated with poor END and prognosis [27,28]. Studies have pointed out that higher HGB has been identified as a specific risk factor for death in type 2 diabetes [29]. HCY has research value in predicting cardiovascular diseases [30] and serum HCY is correlated with post-stroke epilepsy and cognitive function [31]. Although the authors did not observe diabetes as a risk event for END in patients, the present study points to HGB and HCY as risk factors for END.

Among stroke subtypes, large atherosclerotic thrombosis accounts for the majority [32]. In this study, 47.5 % of the 200 patients had atherosclerotic thrombosis. The decrease or lack of sDKK3 level may accelerate atherosclerosis and exacerbate the formation of new intima, which may also negatively affect the functional outcome after AIS [33] In addition, DKK3 is involved in the proliferation, differentiation, and survival of nerve cells and changes in cerebral blood flow, especially in the context of acute or gradual reduction of cerebral perfusion [17]. Therefore, sDKK3 also has a certain value in predicting mortality risk in AIS patients. Multivariate risk regression analysis showed that NIHSS at admission, admission to IUC, and sDKK3 below the threshold (93.0 pg/mL) were independent risk factors for death.

## Conclusion

Serum KDD3 in patients with AIS within 24 hours after admission may be positively correlated with END. Serum DKK3 (93.0 pg/mL) levels can be used to distinguish AIS patients with END from those without END. DKK3 has the potential to be a promising biomarker for progressive adverse events in this patient population.

## Declaration of competing interest

The authors declare conflicts of interest.
